# Bcl-xL mediates therapeutic resistance of a mesenchymal breast cancer cell subpopulation

**DOI:** 10.18632/oncotarget.2634

**Published:** 2014-11-28

**Authors:** Ulrike Keitel, Andreas Scheel, Jürgen Thomale, Rovena Halpape, Silke Kaulfuß, Christina Scheel, Matthias Dobbelstein

**Affiliations:** ^1^ Institute of Molecular Oncology, Göttingen Center of Molecular Biosciences (GZMB), Faculty of Medicine, University of Göttingen, Göttingen 37077, Germany; ^2^ Institute of Pathology Nordhessen, Kassel 34119, Germany; ^3^ Institute of Cell Biology (Cancer Research), Faculty of Medicine, University Duisburg-Essen, Essen 45122, Germany; ^4^ Institute of Human Genetics, University Medical Center Göttingen, Göttingen 37073, Germany; ^5^ Whitehead Institute for Biomedical Research, Cambridge, MA 02142, USA; ^6^ Institute for Stem Cell Research, Helmholz Center Munich, Neuherberg 85764, Germany

**Keywords:** Bcl-xL, chemo-resistance, epithelial-mesenchymal transition, apoptosis, BH3-mimetic

## Abstract

The transition from an epithelial to a mesenchymal phenotype (EMT) confers increased invasiveness and clonogenic potential to tumor cells. We used a breast epithelium-derived cell culture model to evaluate the impact of EMT on the cellular sensitivity towards chemotherapeutics and apoptotic stimuli. Cells that had passed through an EMT acquired resistance towards chemotherapeutics and death ligands. Mechanistically, we found that the levels of the apoptosis inhibitor Bcl-xL were strongly enhanced in mesenchymal versus epithelial cells, whereas the pro-apoptotic proteins Bim and Puma were diminished. Clinical samples from breast cancer showed enhanced Bcl-xL staining in cells that had dispersed into the desmoplastic stroma, as compared to cells that were part of large tumor cell aggregates, suggesting increased Bcl-xL expression when cells invade the stroma. Bcl-xL was necessary for apoptotic resistance in mesenchymal cells, and its expression was sufficient to confer such resistance to epithelial cells. To antagonize Bcl-xL, BH3-mimetics were used. They successfully interfered with the proliferation and survival of mesenchymal cells, and also inhibited the growth of xenograft tumors raised from the mesenchymal subpopulation. We conclude that enhanced Bcl-xL levels confer resistance to cells upon EMT, and that Bcl-xL represents a promising target for therapy directed against invasive cancer cells.

## INTRODUCTION

Epithelial-Mesenchymal Transition (EMT) describes a gene-regulatory cascade that drives epithelial cells towards a more mesenchymal phenotype [1]. When carcinoma cells undergo EMT, they gain the ability to invade the adjacent healthy tissue [2]. Remarkably, EMT also confers properties of stem cells, leading to markedly enhanced clonogenic potential [3, 4]. EMT can be induced by the activation of transcription factors such as Twist and Snail and/or by signaling pathways initiated by Wnt ligands, TGF-beta and others. EMT is characterized by the disappearance of epithelial marker genes such as CD24 or E-cadherin, and by the upregulation of mesenchymal markers, e. g. CD44, Vimentin, and N-cadherin.

Tumors that have undergone EMT may acquire resistance to chemotherapy. A correlation was identified between EMT and resistance to Doxorubicin in the human breast cancer cell lines MDA-MB-231 and BT-549 [5]. Epithelial mouse mammary carcinoma cells (MMC) gained resistance to Oxaliplatin, Taxol and Etoposide upon EMT [6]. Other cell types, including epithelial colon carcinoma cell lines or ovarian cancer cells, acquired resistance to chemotherapeutic agents, in parallel to the occurrence of mesenchymal properties as well [7, 8]. The invasive subpopulations of MCF-7 and MDA-MB-434 cells that have undergone EMT displayed increased levels of Twist which was associated with Paclitaxel resistance [9]. Conversely, the reduction of Twist in breast cancer cells resulted in a partial chemosensitization [10, 11]. These observations all point to the question by what mechanism EMT leads to chemoresistance.

A model of EMT is provided by immortalized and/or transformed human breast epithelial cells (Human Mammary Large T-antigen immortalized Epithelial cells; HMLEs; when expressing haRAS, this is reflected by the designation HMLE RAS; [12, 13]. These cells contain a mesenchymal subpopulation (MSP) characterized by a CD44^high^/CD24^low-negative^ population [3]. Mesenchymal cells were also derived from HMLE cells by a combination of paracrine factors and pertubation of cell-cell adhesion [13]. Apart from enhanced migration and invasion, some of the MSP cells also display a stem cell-like phenotype with enhanced clonogenicity and tumor formation [3]. Moreover, MSP cells respond poorly to a variety of cytotoxic drugs. Only a few drugs were identified to eliminate MSP cells with at least similar efficacy as their epithelial counterparts, namely salinomycin [14] and a number of inhibitors that interfere with Protein Kinase C alpha [15]. In both cases, the exact molecular mechanism of how these drugs confer death to the mesenchymal cell subpopulation remains to be fully elucidated. Even more importantly, the question remains why MSP RAS cells are resistant to multiple cytotoxic compounds.

Here we show that MSP RAS cells contain high levels of the anti-apoptotic protein Bcl-xL, rendering them resistant to treatment with a panel of chemotherapeutics (cisplatin, carboplatin, doxorubicin, neocarzinostatin) as well as death ligands (Trail, TNFalpha). Importantly, through knockdown in MSP cells as well as overexpression in parental HMLE cells, we show that Bcl-xL is necessary and sufficient for this resistant phenotype of HMLE cells. Moreover, targeting Bcl-xL by BH3-mimetics interferes with survival of epithelial as well as mesenchymal cell populations and also reduced the growth of RAS-transformed MSP cells in tumor xenografts. Together, these results outline a strategy to overcome tumor heterogeneity by reducing cytotoxic resistance in both epithelial and mesenchymal cancer cells. Importantly, Bcl-xL-overexpression was also observed in N-Cadherin-positive, singly migrating breast cancer cells at the invasive front in clinical samples, suggesting that the association of Bcl-xL overexpression and EMT might be a common occurrence in breast carcinoma.

## RESULTS

### Upon EMT, breast epithelial cells gain resistance towards chemotherapy and death ligands

To study the impact of EMT on the response to cancer drugs, we used MSP cells that had been sorted from the bulk of HMLEs (24^+^ HMLE and 24^+^ RAS) as described previously [13]. This cell population displayed all the characteristics of mesenchymal cells, including the expression of mesenchymal marker genes and the suppression of epithelial markers [13]. This was true in native and RAS-transformed HMLEs, as revealed by array hybridization and quantitative RT-PCR (Supplemental Fig. S1A-B and Supplemental Table 1).

**Figure 1:**
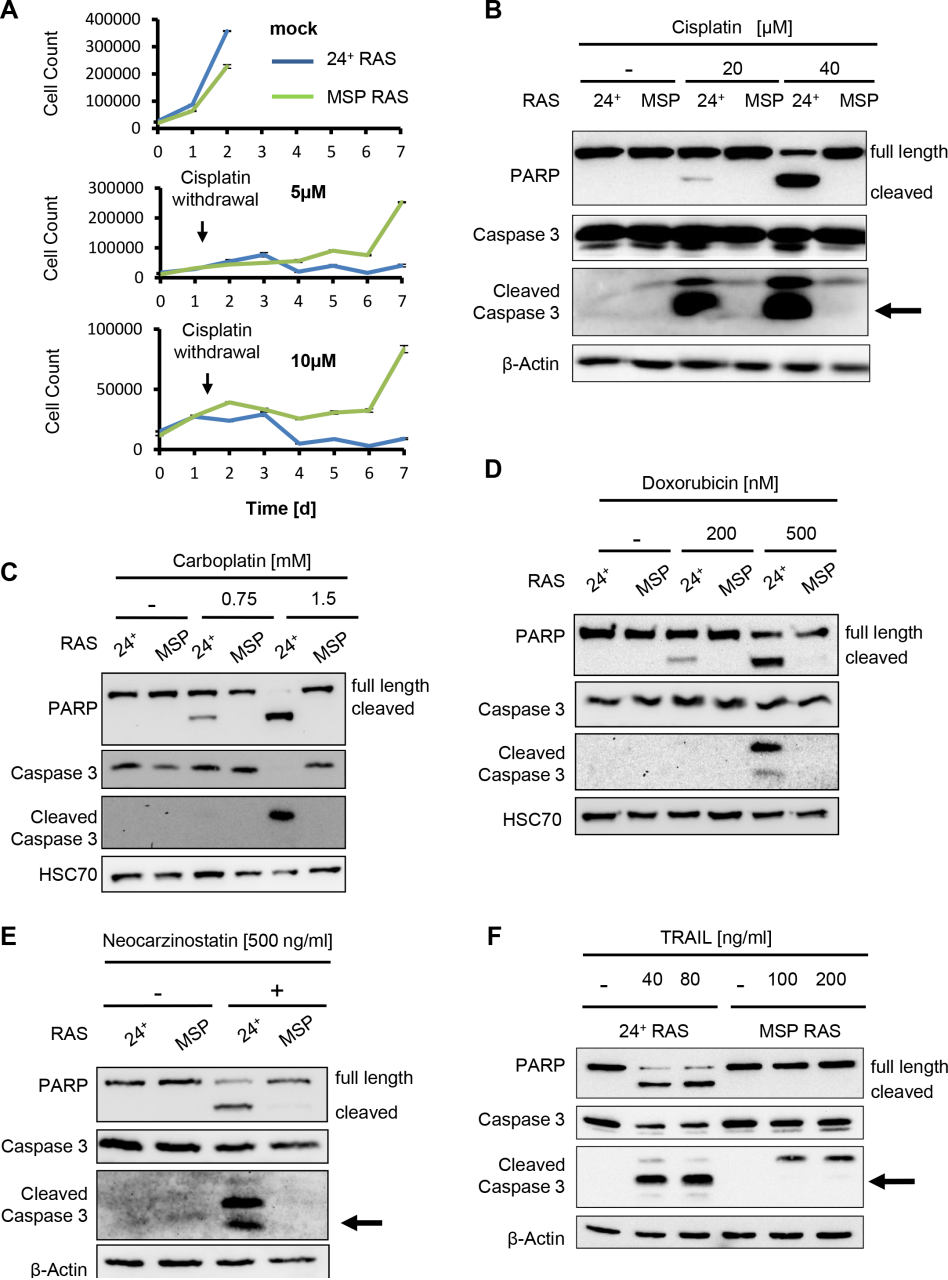
MSP RAS cells resist chemotherapeutic treatment and apoptosis induction **(A)** HMLE RAS cells were treated with 5μM or 10μM cisplatin, for 24h, followed by further incubation without chemotherapeutics. The cell number was determined daily for 7d, using automated light microscopy with quantitative image analysis (Celigo cytometer). Non-treated cells were used as controls. **(B-F)** HMLE RAS cells were treated with the indicated concentrations of cisplatin (B) or carboplatin (C), each for 16h, with doxorubicin for 24h (D), with neocarzinostatin for 20h (E) or Trail in the presence of 20μg/ml cycloheximide for 6h (F). Controls were treated with corresponding amounts of the respective solvent. Cell lysates were analysed by immunoblotting. β-Actin or HSC70 staining was used as loading control.

We fully transformed these cells by expression of ha-RAS [12] and then assessed the susceptibility of epithelial, CD24 positive (24^+^) HMLE RAS cells (referred to 24^+^ RAS cells) and MSP RAS cells to chemotherapeutics. Upon treatment with cisplatin, MSP RAS cells survived to a far greater extent than their epithelial counterparts 24^+^ RAS cells (Fig. 1A), and they also showed a much lower extent of PARP cleavage (indicative for caspase activation; Fig. 1B). We determined the extent to that the cellular DNA was modified by cisplatin by measuring the levels of intrastrand platin adducts, but did not observe significant differences between 24^+^ RAS and MSP RAS cells (Supplemental Fig. S1C). This largely excludes differences in drug transport or metabolism as a possible mechanism of MSP RAS cell resistance. Moreover, similar resistance of MSP RAS cells was found when treating the cells with carboplatin (Fig. 1C), the topoisomerase inhibitor doxorubicin (Fig. 1D), and the radiomimetic neocarzinostatin (Fig. 1E). The lack of caspase activation under these circumstances prompted us to test whether MSP RAS cells may also be resistant to treatment with the death receptor ligands Trail (Fig. 1F) and TNF alpha (Supplemental Fig. S1D), and this was indeed the case. Further, performance of cell viability assays revealed a better overall survival for MSP RAS cells compared to 24^+^ RAS cells, upon triggering cell death with chemotherapeutics (Supplemental Fig. S1E).

We conclude that EMT renders cells resistant not only towards chemotherapeutics, but also towards DNA-damage independent apoptotic stimuli. Thus, it appears conceivable that cells that have passed through an EMT are generally resistant to induction of apoptosis.

### Bcl-xL levels are enhanced upon EMT

**Figure 2:**
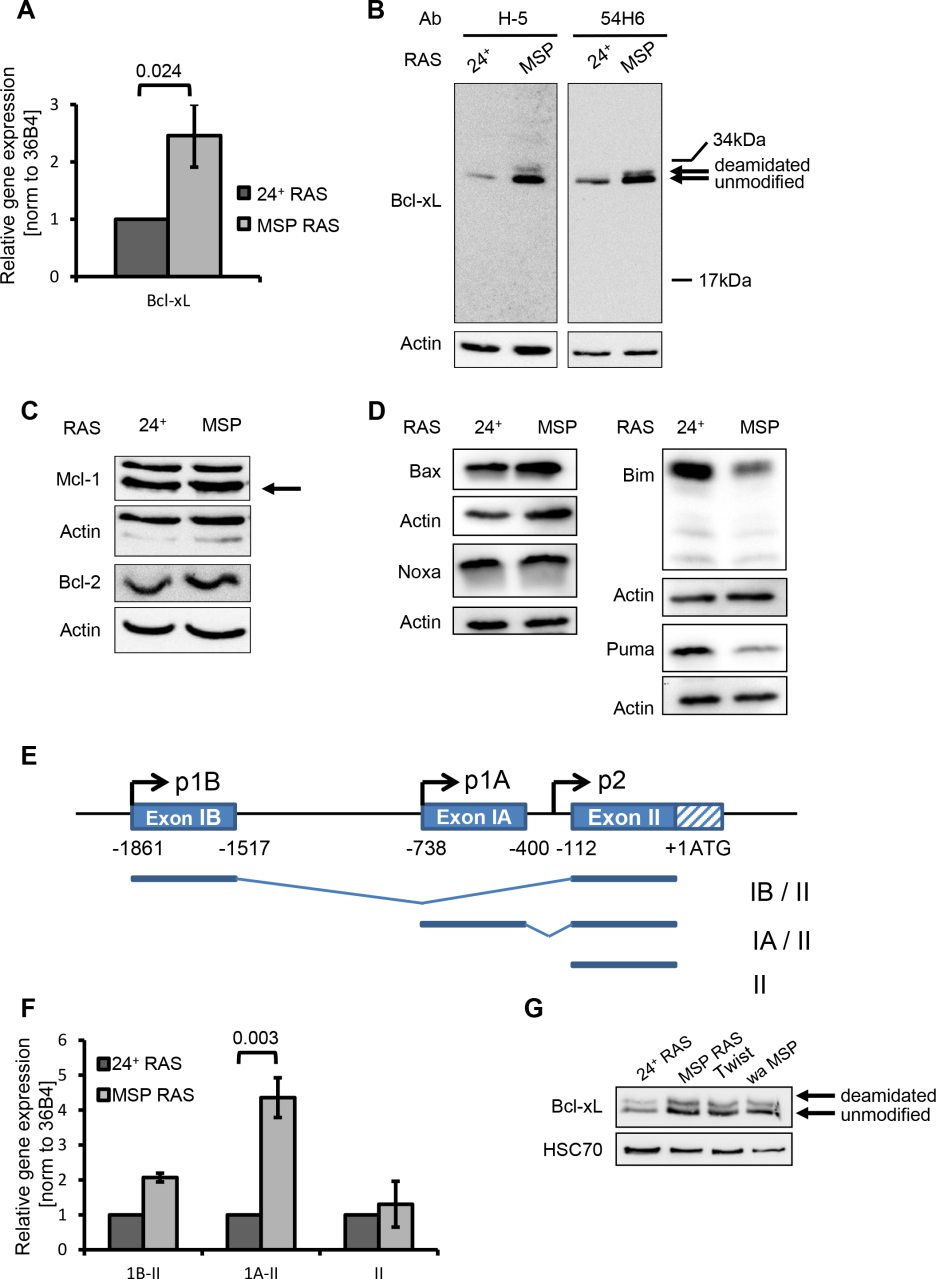
EMT enhances the levels of the anti-apoptotic protein Bcl-xL and diminishes the levels of the pro-apoptotic proteins Bim and Puma **(A)** mRNA encoding Bcl-xL was quantified by qRT-PCR. **(B-D)** Protein lysates were analysed to detect Bcl-xL (B), other anti-apoptotic (C) or pro-apoptotic (D) Bcl2-familiy members by immunoblotting. Bands corresponding to deamidated or unmodified Bcl-xL [39] are indicated by arrows. **(E)** Schematic presentation of the *BCL2L1* gene with alternate promoters. (Top) The distal (IB) and proximal (IA) non-coding exons, and part of the first coding exon (II) including the translational start site (ATG). Additionally, the three described *BCL2L1* promoters (p1B, p1A, p2) are depicted [17]. (Bottom) Major BCL2L1 transcripts starting from promoter p1B or p1A, comprising exon IB or IA, respectively, or starting upstream from exon II. **(F)** BCL2L1 mRNA transcripts were analysed by qRT-PCR using primers that specifically span exons I – II, IA – II, or II alone, respectively. These mRNA levels were normalized to that of 36B4 mRNA. Columns and error bars represent the mean ± S.E.M. of *n* = 3. **(G)** Bcl-xL was detected in 24^+^ RAS and MSP RAS cells, compared with mesenchymal cell populations that had been obtained by Twist overexpression (Twist), or by limited trypsinization based on their weak adherence (wa MSP).

When comparing the expression patterns of 24^+^ and MSP cells by array hybridization (Supplemental Table 1), we noticed enhanced expression of the *BCL2L1* gene in RAS-transformed and native MSP cells. This was confirmed by quantitative RT-PCR analysis (Fig. 2A). *BCL2L1* gives rise to the anti-apoptotic gene product Bcl-xL, but also to the isoform Bcl-xS that antagonizes Bcl-xL functions [16]. mRNAs corresponding to both isoforms were augmented in MSP RAS cells (Supplemental Fig. S2A). However, when performing immunoblot analyses with two different antibodies predicted to bind either both isoforms or the large one, respectively, only one protein with a molecular weight corresponding to Bcl-xL was detected, with stronger band intensities in MSP RAS compared to 24^+^ cells (Fig. 2B). We conclude that the Bcl-xL protein is the predominant *BCL2L1* gene product in HMLE cells and that its levels are enhanced in the MSP cells. In contrast, other anti-apoptotic regulators of the intrinsic apoptotic pathway, Mcl-1 and Bcl-2, did not differ in their levels between epithelial and mesenchymal cell populations (Fig. 2C). However, the pro-apoptotic Bcl-2 family members Bim and Puma seemed to be diminished in their protein levels in MSP RAS cells, which can additionally sustain apoptosis-resistance upon EMT (Fig. 2D).

The *BCL2L1* gene has several transcription start sites (Fig. 2E), giving rise to mRNAs with different 5′ ends. When performing RT-PCRs to determine the levels of each transcript, we found the mRNA driven by the second promoter (designated “1A” in previous literature [17]) to be particularly enhanced in MSP cells (Fig. 2F). Thus, we propose that the levels of Bcl-xL are increased in MSP cells through activation of the 1A promoter of *BCL2L1*.

Bcl-xL levels were also increased in MSP cells that were isolated based on their weaker adherence (wa MSP), and in HMLE Twist cells which have undergone EMT as a consequence of Twist overexpression. Thus, EMT induced Bcl-xL expression regardless of the stimulus that had led to EMT in the first place (Fig. 2G). The up-regulation of Bcl-xL levels protects these cell lines from apoptosis induction upon cisplatin treatment as is was seen for MSP RAS cells before (Supplemental Fig. S2B).

### Bcl-xL levels are enhanced in breast carcinoma cells that invade stromal tissue

**Figure 3:**
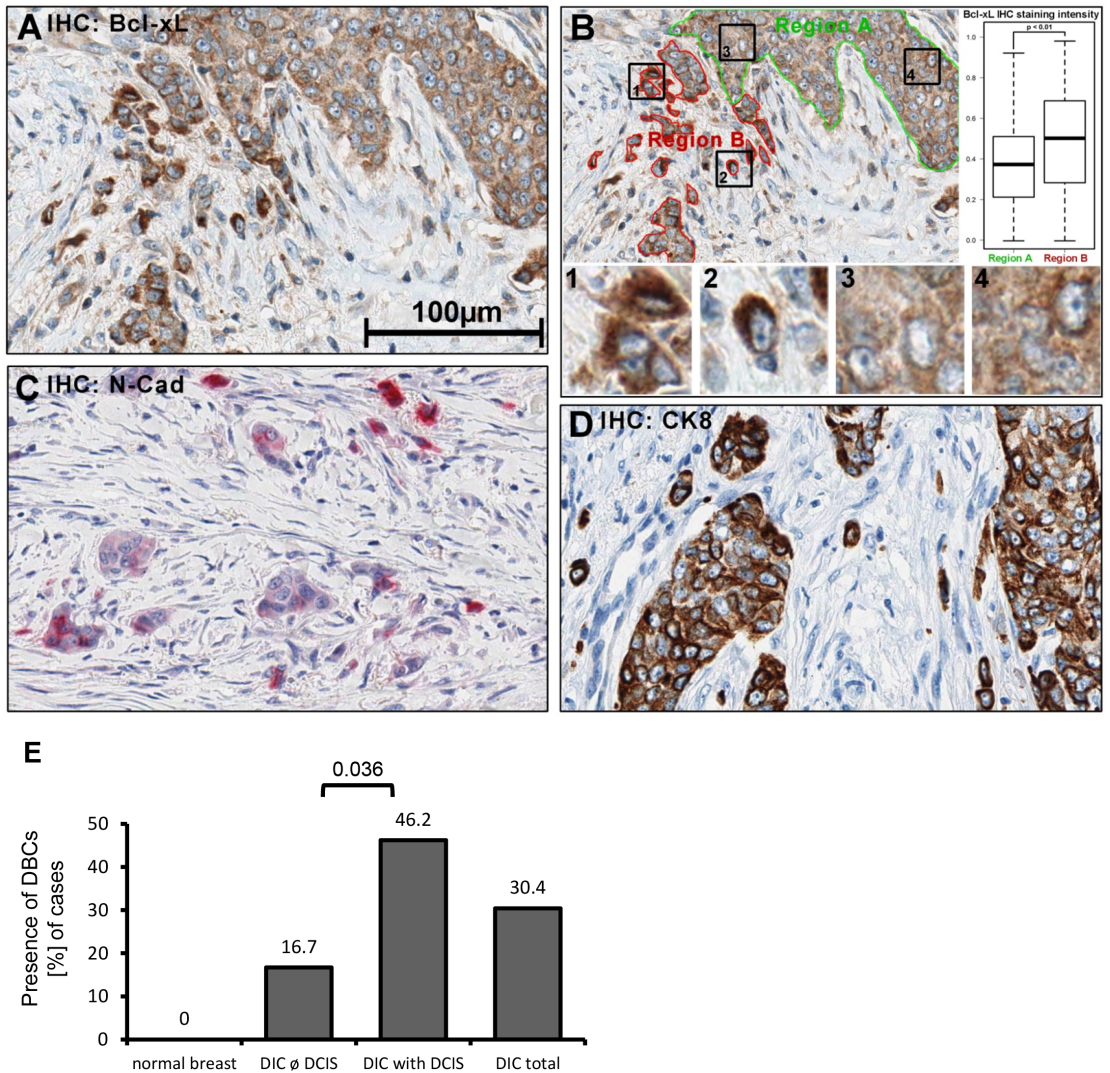
Bcl-xL protein levels are increased in human breast cancer cells that are dispersed in the desmoplastic stroma Immunohistochemistry (IHC) of human tissue, fixed in PBS-buffered formalin (4%), was embedded in paraffin. 1.5μm sections were treated with boric-acid/EDTA buffer for antigen-retrieval, followed by incubation with primary antibodies, secondary peroxidase-coupled antibodies, and diaminobenzidine (DAB). A more extensive array of staining is shown in Supplemental Fig. S3. **(A)** Detection of Bcl-xL in breast cancer samples. **(B)** Quantification of DAB precipitate color-intensity in the two highlighted regions (borders green, red); boxplots of respective intensities. 1′-′4′: Magnified details as indicated. Statistical testing was performed using Student´s T-test. **(C)** N-cadherin IHC (Fast Red). **(D)** Cytokeratin 8 IHC. B-D: 400x magnification. **(E)** Presence of dispersed, Bcl-xL enhanced cells (DBCs) in correlation to the presence and absence of ductal carcinoma in situ (DCIS), *n* = 56.

Predominantly in experimental models, EMT has been demonstrated to be a major driving force in cancer cell invasion [18]. We therefore sought to assess whether enhanced Bcl-xL levels can also be found in cancer cells at the invasive front of breast carcinomas. A collection of fifty-six breast cancer specimens was examined by immunohistological staining of tissue sections against Bcl-xL. Indeed, we observed moderate-to-strong Bcl-xL staining in a majority of the samples (*n* = 46, 82%). However, the strongest signal was obtained in invasive cancer cell subpopulations that were surrounded by stromal cells, as confirmed by quantitative morphometric analysis of the staining pattern. Specifically, single or small cell clusters of strongly Bcl-xL staining cells were found within the desmoplastic stroma and its fibroblasts (Fig. 3A, Supplemental Fig. S3A), presumably representing the forefront of tumor cell invasion. These dispersed, Bcl-xL enhanced cells (DBCs) not only showed strong cytoplasmic staining for Bcl-xL, but the staining intensity was consistently enhanced when compared to continuous clusters of tumor cells on the same section (Fig. 3B). Interestingly, 46% of all investigated cases of ductal invasive carcinoma (DIC) featuring an *in situ* component (ductal carcinoma in situ, DCIS) contained DBCs compared to 16% tumors entirely consisting of invasive carcinoma (DIC) (Fig. 3E, *p* = 0.036). Importantly, the DBCs also displayed enhanced staining for N-cadherin, a mesenchymal marker, supporting the notion that these singly or in small clusters migrating DBCs have undergone an EMT (Fig. 3C-D; Supplemental Fig. S3B-C). Additionally, we noticed a significant (*p* = 0.004) association between αER^+^/Her2^−^ staining pattern and the presence of DBCs (Supplemental Table 2.1). There was no significant correlation of DBC appearance with other parameters such as histopathological grading, tumor size, nodal status and distant metastasis (Supplemental Table 2.2).

In normal breast epithelium, we observed a light staining of Bcl-xL that was restricted to the luminal layer, supporting the notion that Bcl-xL might be characteristic for breast cancer with luminal features (Supplemental Fig. S3D). Taken together, these data suggest that a distinct subpopulation of tumor cells, preferentially in an ER^+^ DIC with a DCIS component, undergoes EMT, invades the desmoplastic stroma, and simultaneously acquires apoptotic resistance through Bcl-xL-expression. More studies are warranted to determine the universality of this mechanism: however, the fact that the HMLE cell culture model reflects basal-like breast carcinoma [19] may suggest that EMT and Bcl-xL overexpression can be associated irrespective of tumor type.

### Bcl-xL levels are critical for apoptotic susceptibility before and after EMT

**Figure 4:**
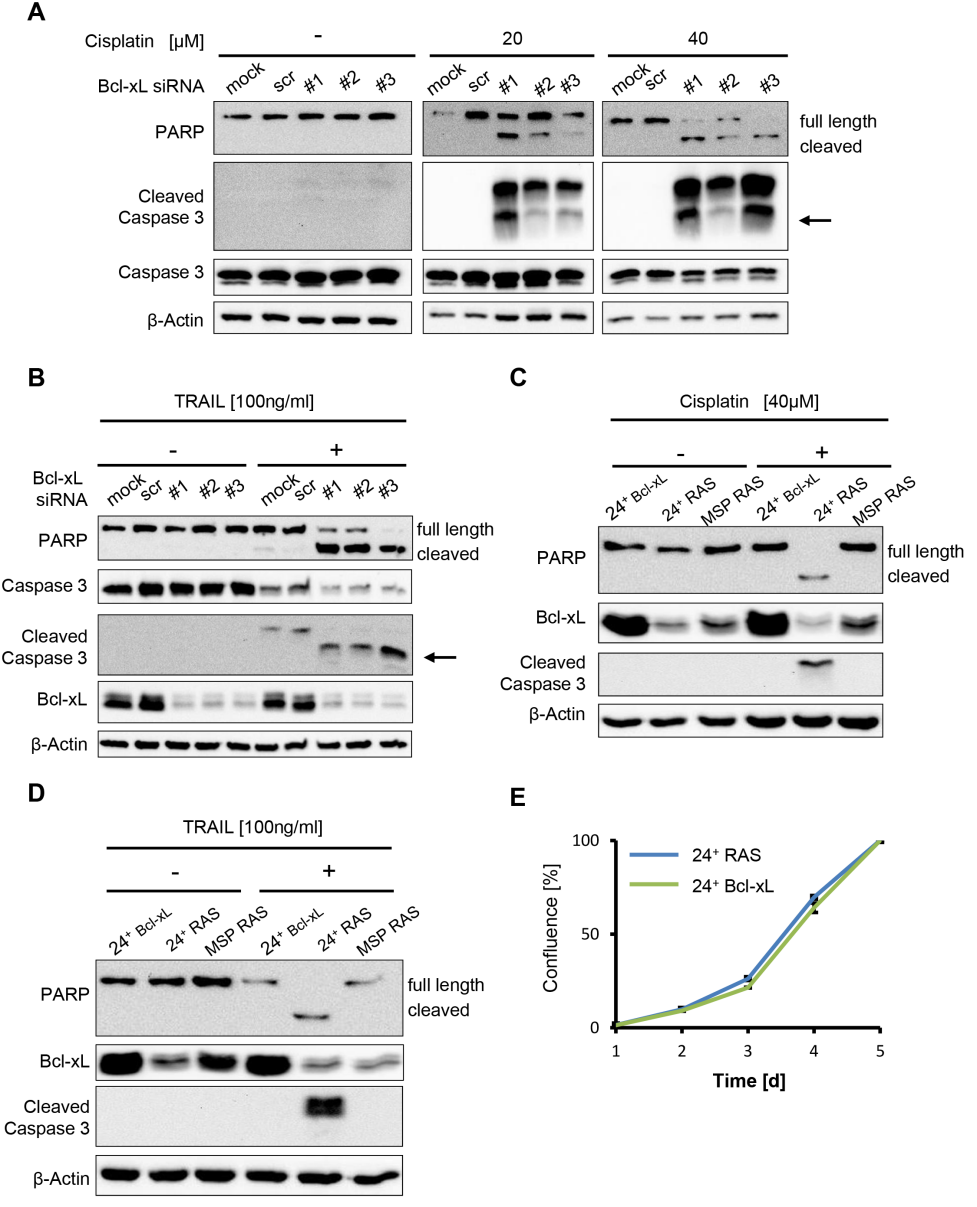
Bcl-xL levels determine apoptosis but not cell proliferation in HMLE RAS cells **(A, B)** MSP RAS cells were depleted of Bcl-xL by siRNA and treated with 20μM and 40μM cisplatin for 16h (A), or with 100ng/ml Trail in the presence of 20μg/ml of the inhibitor of protein synthesis cycloheximide (CHX) for 6h (B). Cell lysates were analysed by immunoblotting. Untreated (mock) and scrambled (scr) siRNA-transfected cells were used as controls (see also Supplemental Fig. S4B). An arrow is indicating the molecular weight of cleaved caspase 3 (17kDa). A longer exposure for non-treated cells was used to ensure that cleaved Caspase 3 would have been detected if present. **(C, D)** 24^+^ Bcl-xL and HMLE RAS cells were treated with 40μM cisplatin for 16h (C) or 100ng/ml Trail in the presence of 20μg/ml CHX for 6h (D), followed by immunoblot analysis. **(E)** Proliferation of 24^+^ RAS and 24^+^ Bcl-xL overexpressing cells was monitored for 5d. Cell confluence was determined daily using automated light microscopy with quantitative image analysis (Celigo cytometer).

Since enhanced Bcl-xL levels accompany EMT in HMLE and HMLE RAS cells as well as in clinical breast cancer samples, we next determined whether Bcl-xL is actually a determinant of cell survival and chemoresistance in this context. To test this, we depleted both the elevated as well as lower levels of Bcl-xL in epithelial and mesenchymal HMLE cells, respectively, by siRNA transfection, followed by cisplatin treatment, and we determined the onset of apoptosis by detecting cleaved PARP and activated Caspase 3. In the control transfections (mock or control siRNA), MSP RAS cells were again found to resist cisplatin exposure, while the epithelial 24^+^ RAS cell population was highly sensitive. In contrast, however, when depleting the cells of Bcl-xL, this difference was completely abolished, and both cell populations activated caspases when treated with cisplatin (Fig. 4A; Supplemental Fig. S4A). Thus, elevated Bcl-xL expression is necessary for cisplatin resistance of MSP and MSP RAS cells. Moreover, Bcl-xL depletion by siRNA also rendered MSP RAS cells sensitive to the death ligand Trail, suggesting that elevated Bcl-xL levels are necessary for general resistance to apoptotic stimuli (Fig. 4B). Conversely, we engineered HMLE cells to overexpress Bcl-xL by lentiviral transduction. Doing so prevented caspase activation by cisplatin (Fig. 4C) and Trail (Fig. 4D) in otherwise sensitive 24^+^ RAS cells. In contrast, we exclude that 24^+^ RAS cells with normal Bcl-xL levels and 24^+^ Bcl-xL overexpressing cells displayed difference in their proliferation (Fig. 4E). Therefore, taken together we conclude that Bcl-xL is both necessary and sufficient to determine the sensitivity of HMLE cells and their mesenchymal derivatives towards chemotherapy as well as death ligands.

### BH3-mimetic drugs interfere with the proliferation of epithelial as well as mesenchymal cell populations

**Figure 5:**
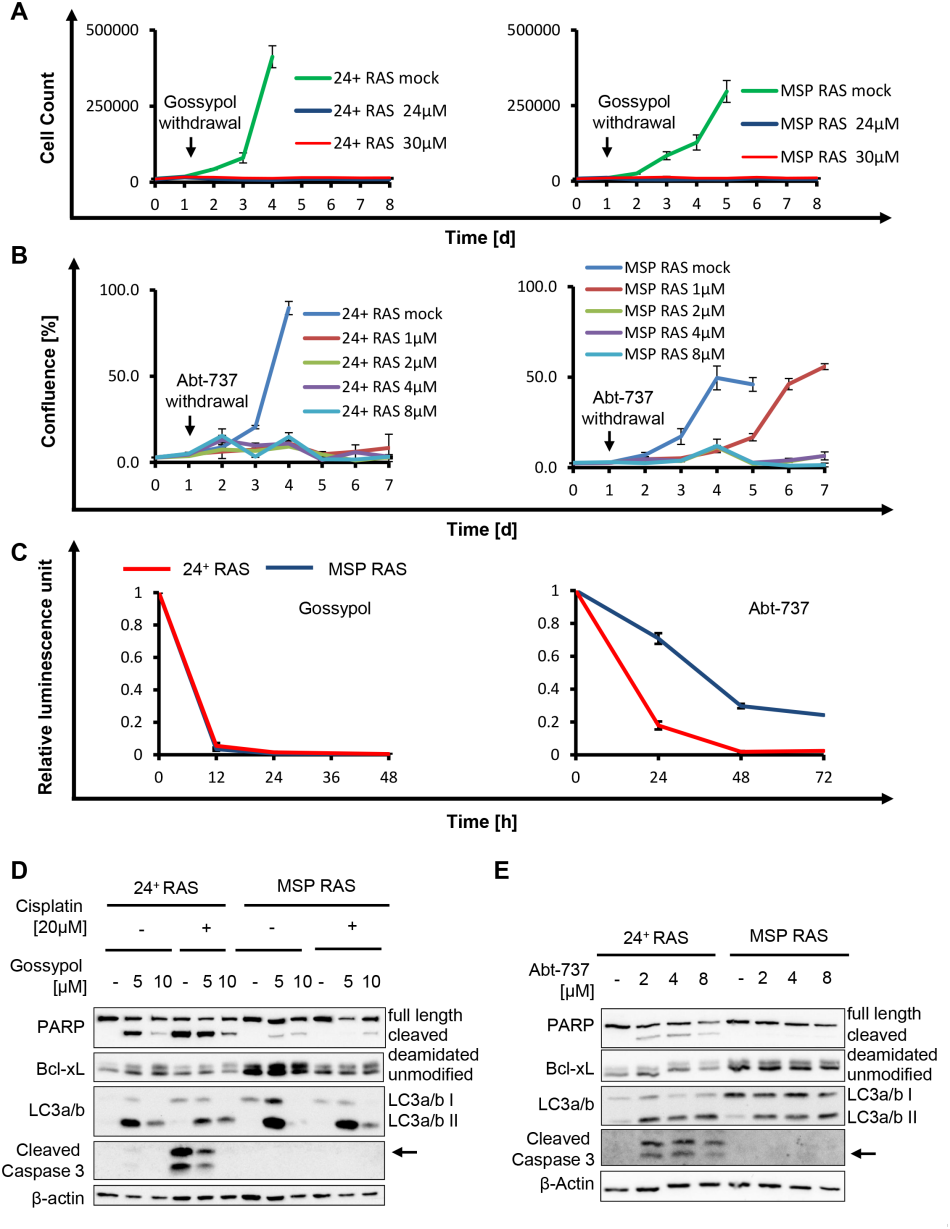
The BH3-mimetics gossypol and Abt-737 overcome apoptosis resistance of MSP RAS cells **(A, B)** HMLE RAS cells were treated with gossypol (A) or Abt-737 (B) for 24h, followed by further incubation without the drug. DMSO was used as control. Cell number or cell confluence was measured daily for 8d and 7d with a Celigo cytometer, respectively. **(C)** HMLE RAS cells were treated with 10μM Gossypol or 10μM Abt-737 for 48h and 72h, respectively. Cell viability upon treatment was determined by assessing the ATP concentration in cell lysates using a luciferase assay. **(D)** HMLE RAS cells were exposed to gossypol, or DMSO, in the presence or absence of 20μM cisplatin, for 16h. **(E)** HMLE RAS cells were treated with Abt-737 for 16h. Cell lysates were analysed by immunoblotting. LC3a/b II served as an indicator for autophagy. During autophagy, the cytosolic LC3a/b I (16kDa) is converted to the autophagosome-associated LC3a/b II (14kDa) through lipidation [27].

**Figure 6:**
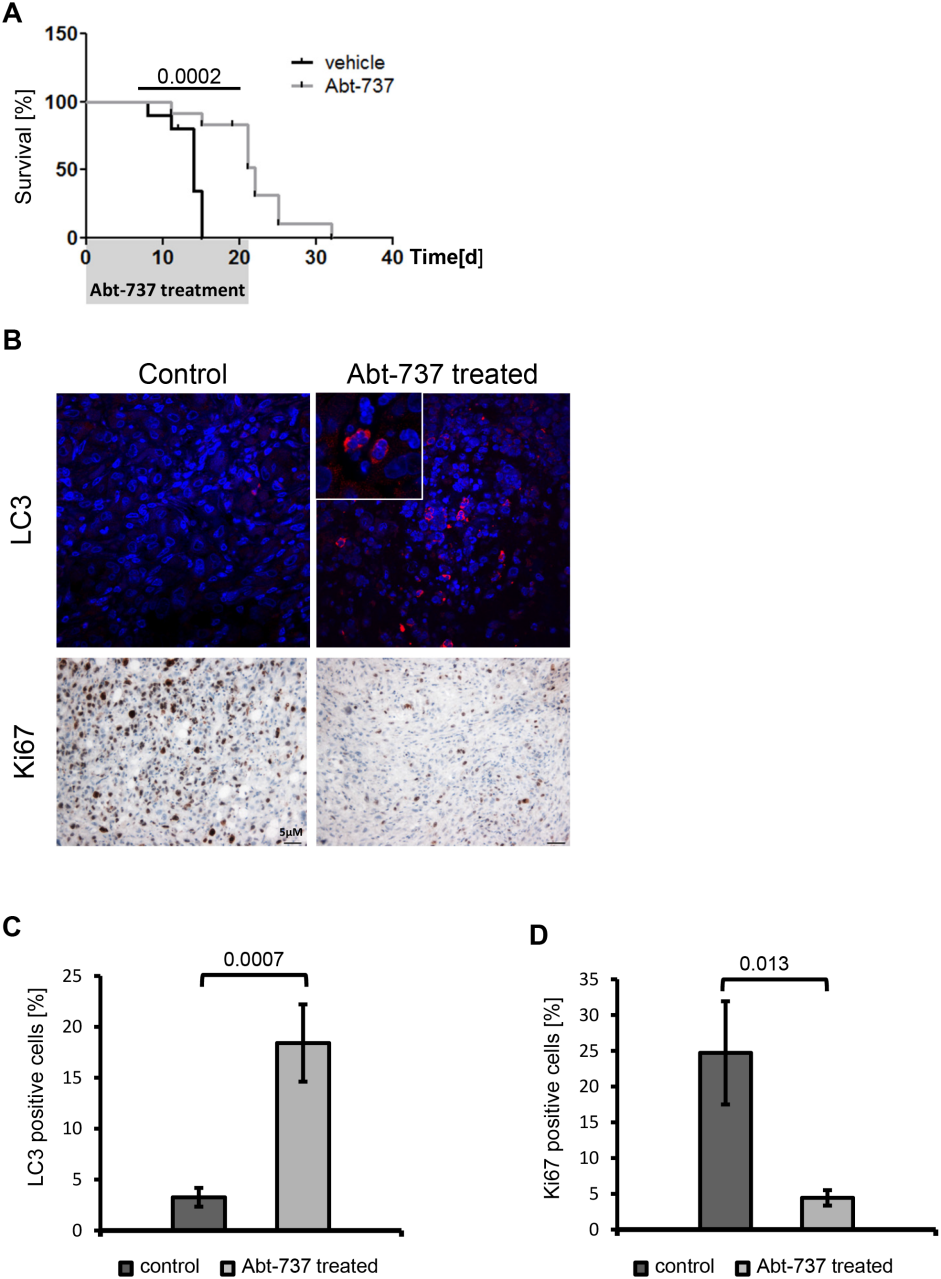
BH3-mimetics prolong the survival of mice carrying MSP RAS xenografts **(A)** 10^6^ MSP RAS cells were subcutaneously injected in (Nu/Nu) mice to form a tumor xenograft. Three weeks thereafter, the mice were treated with intraperitoneal injections of 75mg/kg/day Abt-737 (light grey line, *n* = 10) or vehicle (dark line, *n* = 9) for 21d. The mice were euthanized when a tumor size of 15mm in diameter had been reached. The shaded area corresponds to the duration of treatment. **(B)** IHC and IF of xenograft tumor tissue for detection of Ki67 or LC3, respectively, in control and Abt-737 treated mice. 600x magnification for LC3, inlet 4x zoom. 100x magnification for Ki67. A more extensive array of stainings is shown in Supplemental Fig. S6. **(C)** Quantification of LC3 positive cells across all treated vs. mock-treated tumors. **(D)** Quantification of Ki67 positive cells. Columns and error bars represent the mean ± S.E.M.

Finally, we interfered with Bcl-xL activity pharmacologically, as a strategy to eliminate tumor cells including the mesenchymal subpopulation. The BH3-mimetic drug gossypol [20–22] and its derivative Abt-737 [23–25] were used to treat each 24^+^ RAS and MSP RAS cells. As a result, both epithelial and mesenchymal HMLE RAS stopped proliferating (Fig. 5A-B and Supplemental Fig. S5). Somewhat higher doses of Abt-737 were needed to reduce the proliferation of the mesenchymal subpopulation, perhaps reflecting the higher levels of the drug target Bcl-xL. On the other hand, no such difference was seen in the case of gossypol. Treatment with BH3-mimetic drugs also reduced cell viability of both cell populations (Fig. 5C). Interestingly, autophagy rather than classical apoptosis appeared as the major cause of cell death in response to BH3-mimetic treatment. This was revealed by the accumulation of microtubule-associated protein 1A/1B-light chain 3 II (LC3a/b II), (Fig. 5D-E) reflecting the lipidation of cytosolic LC3 (LC3a/b I) by an ubiquitin-like system forming LC3a/b II in association with autophagosomal membranes [26, 27]. Possibly, BH3-mimetics inhibit the interaction between Bcl-xL and Beclin1 at the ER, thereby inducing autophagy [28]. We then assessed the *in vivo* efficacy of Abt-737 in a mouse tumor model derived from MSP RAS cells. Abt-737 treatment prolonged the survival of tumor-bearing animals to a highly significant degree, compared with DMSO-treated animals (Fig. 6A). In accordance with our findings *in vitro*, xenograft tumors that were treated with Abt-737 exhibit significantly (*p* = 0.0007) increased amounts of LC3 positive cells compared to the non-treated tumors (Fig. 6B, C and Supplemental Fig. S6A-B). In contrast, proliferation was significantly (*p* = 0.013) reduced upon Abt-737 application as determined by Ki67 staining (Fig. 6B, D). Taken together, the use of BH3 antagonists represents a promising strategy to target both epithelial breast cancer cells and their mesenchymal, apoptosis-resistant derivatives.

### DISCUSSION

Our results strongly suggest that the upregulation of a specific Bcl-xL transcript renders breast cancer cells that have passed through an EMT resistant towards apoptotic stimuli. We propose that Bcl-xL upregulation represents a mechanism that renders invasive cancer cells resistant to a large variety of cytotoxic agents. A pleiotropic program directing cell fate decisions, EMT thus determines at least three major components of the cellular phenotype: invasion and metastasis; stemness and clonogenicity; and resistance towards death stimuli.

We demonstrated that our findings are not only valid for a cell culture model, but are also applicable to clinical samples of human breast cancer tissue. Previous investigations of invasive breast cancer suggested the association of Bcl-xL with higher tumor grade and decreased overall survival, but, importantly, did not report on the intratumoral distribution and the heterogeneity of Bcl-xL-expressing cells that we observed and quantified [29]. In the present study, we have shown that Bcl-xL protein levels are heterogenously distributed within human breast cancer tissues, and specifically elevated in dispersed cells, invading singly or in small clusters into the desmoplastic stroma surrounding the tumor. Supporting the notion that these dispersed, Bcl-xL overexpressing cells (DBCs) have undergone an EMT, we observed a striking correllation between the localization of these cells in the stroma, Bcl-xL overexpression and a strong N-Cadherin staining (as a marker of mesenchymal differentiation). Although pathologic specimens can only provide a snapshot-like image without immediate functional assessment, the presence of single cancer cells or small groups of cancer cells, surrounded by desmoplastic stroma, at least suggests that these cells represent the forefront of cancer invasion and may even seed distant metastases. Hallmarks of EMT in tumor cells at the invasive front were previously described [30–32] and we now show that such cells increase the levels of a key anti-apoptotic factor. The combined enhancement of N-cadherin and Bcl-xL further supports the view that EMT-induced Bcl-xL characterizes this particularly aggressive subpopulation of human cancer cells.

One could speculate that the enhanced levels of Bcl-xL in the course of EMT may actually represent a cause of the mesenchymal or stem-like phenotype. However, when stably overexpressing Bcl-xL in the epithelial cell population, we did not observe enhanced spontaneous EMT (data not shown). Though these data suggest that Bcl-xL upregulation might be a consequence, but not a cause of EMT, further studies are warranted to determine whether Bcl-xL upregulation sensitizes cells to EMT-inducing stimuli.

Out data reveal Bcl-xL inhibition as an attractive strategy to target actively invading tumor cells. And indeed, Bcl-xL can be targeted pharmacologically. The most direct way of doing so consists in the use of BH3-mimetics. Our results suggest that BH3-mimetics may be suitable to not only target a major mechanism of cell survival, but to overcome the resistance mechanisms that would otherwise allow cancer stem cells to withstand a large variety of cancer therapeutic regimens. Several clinical phase II studies for gossypol as well as Abt-737 are registered at the NCI database, suggesting that these reagents may be applicable for a wide range of cancer types and thus representing promising agents for cancer therapy.

BH3-mimetics have targets that are not unique to tumor cells but can be found in most normal cells as well. This raises the question why these drugs still eliminate tumor cells without non-tolerable toxicity towards their normal counterparts. The explanation appears to consist in tumor-associated properties that render the cells addicted to “non-oncogenes” [33]. For instance, most proliferative stimuli, e. g. activation of oncoproteins belonging to the Myc and E2F families, also sensitize these cells to apoptosis, e. g. by activating the p14/Arf – p53 pathway. This renders such cells more susceptible to the inhibition of anti-apoptotic factors such as Bcl-xL, in particular when such targeted drugs are combined with inhibitors of tumor-supportive cellular machineries [34]. Notably, BH3-mimetics were not “selective” for MSP RAS versus epithelial HMLE RAS cells in our experiments. Rather, they showed cytotoxicity against both epithelial *and* mesenchymal cells. This is different from previously described small compounds that preferentially eliminate MSP cells, i. e. Salinomycin [14] and inhibitors of Protein Kinase C alpha [15]. However, we propose that it represents an advantage to target both subpopulations of tumor cells. This is plausible by the phenomenon of tumor cell plasticity, i. e. the intrinsic capability of tumor cells to change from epithelial to mesenchymal and *vice versa* [35]. Indeed, there is mounting evidence that the development from cancer stem cells to their progenitors is not unidirectional but can rather be reversed [36]. As a consequence, a strategy targeting all tumor cells, rather than a specific subpopulation, is predicted to have the highest likelihood of success.

## MATERIALS AND METHODS

### Cell culture, reagents and transfections

Human mammary epithelial cells (24^+^HMLE, MSP, 24^+^ Ras, MSP Ras, HMLE Twist and self-isolated MSP) were cultured in 50% MEGM (Bullet Kit, Lonza), 25% DMEM high glucose and 25% F-12 (both Invitrogen™) media supplemented with 10mg/ml human hydrocortisone, 10mg/ml human insulin and 10μg/ml human EGF (all Sigma-Aldrich). Cisplatin (Neocorp), carboplatin (medac), doxorubicin (diluted in DMSO; Santa Cruz), neocarzinostatin (NCS), cycloheximide (CHX; diluted in Ethanol; both Sigma Aldrich), Trail, TNFalpha (both R&D systems), gossypol (SellectBio), Abt-737 (active biochem, both diluted in DMSO). For siRNA-mediated knock down cells were reverse transfected with 5nM Silencer Select Pre-Designed siRNA or control siRNA (all Ambion) using Lipofectamine 2000 (Invitrogen).

### Isolation of weakly adherent MSP

To generate MSP cells from their parental 24^+^HMLE, limited trypsinization of 24^+^HMLE was performed. Trypsin was applied for exactly 3min at 37°C and 5% CO_2_. Afterwards, the cells detached by rapping on the cell culture flask. The cell suspension containing weakly adherent cells was transferred into a 15ml reaction tube and Trypsin was neutralized by adding Trypsin Neutralizing Solution in the same amount. After centrifugation (8min, 900rcf, RT) the supernatant was removed and the pellet was resolved in supplemented MEGM medium and completely reseeded into a new cell culture flask. The process of limited trypsination was repeated four times every second day to get a complete mesenchymal subpopulation.

### Cell proliferation assay

For cell proliferation analysis, cells were seeded in duplicates into 12-well plates and, after 16h treated with cisplatin, gossypol or Abt-737 in the respective concentrations for 24h. Then, fresh medium without drugs was added. Direct cell counting was performed by defining the cell number per well on the basis of contrast in bright field light microscopy using a Celigo Adherent Cytometer (Brooks Automation). Cell counting or confluence measurement was performed once a day. All proliferation assays were performed in triplicate to ensure the reproducibility of the results.

### Immunoblot analysis

Whole cell lysates were prepared with RIPA lysis buffer (1% Triton X; 1% Desoxycholate, 0,1% SDS; 150 mM NaCl; 10 mM EDTA; 20 mM Tris-HCl; pH 7.5; 100.000KIE Trasylol) freshly supplemented with 2M Urea, Pefablock, 1μg/ml Leupeptin / Aprotinin, 1μg/ml Pepstatin A and 1μM Microcystin. Protein concentration was measured using the BCA protein assay (Thermo Fisher Scientific) and adjusted to equal amounts for all samples. For immunoblotting, protein lysates were separated by SDS polyacrylamide gel electrophoresis and transferred to nitrocellulose membranes, blocked with 5% milk / TBS Tween and incubated with the following antibodies, each diluted in 5% BSA in Tris-buffered saline containing 0,1% Tween20: PARP (C-2-10; Calbiochem), Caspase 3 (8G10), cleaved Caspase 3 (5A1E), Bcl-xL (54H6), Akt, Mcl1 (D35A5), Bcl-2 (50E3), Bax, Bim (C34C5), and LC3a/b (all Cell Signaling Technology). Bcl-xL (H-5) and HSC70 (B-6) (both Santa Cruz), beta Actin (rabbit polyclonal), Noxa (EPR9735B) and Puma (EP512Y, all Abcam). Primary antibodies were detected with peroxidase-coupled secondary antibodies (Jackson). All immunoblot analyses were performed in triplicate to ensure the reproducibility of the results.

### DNA platination assay

Platin-DNA intrastrand crosslinks were detected by immuno-slot-blotting. 100μl extracted DNA of cisplatin-treated cells was denatured for 10min at 95°C cooled on ice and diluted with 100μl ice-cold 2M ammonium acetate. Nitrocellulose Hybond membrane was soaked in 1M ammonium acetate and 200μl sample was applied onto the membrane. The membrane was soaked with 5xSSC for 5min, washed with H_2_O, dried, heated for 2h at 80°C and blocked with 5%milk / TBS Tween. The membrane was incubated with the cisplatin-lesion specific antibody R-C18 (0.2μg/ml) in 5%milk / TBS Tween for 1h at RT [37]. The primary antibody was detected with anti-rat HRP-linked secondary antibody for 1h. All immuno-slot-blot analyses were performed in triplicate.

### Apoptosis assay

Cisplatin-treated cells were stained with the Guava^®^ MultiCaspase FAM Kit (Millipore) to detect active caspases and apoptotic cells by flow cytometry.

### Cell viability assay

1500 cells per well were seeded in triplicate in 96-well plates 1 day before treatment. Cells were treated with drugs and cell viability was determined with the CellTiterGlo Luminescent Cell Viability assay (Promega, Madison, WI, USA). The luciferase assay determines the remaining amount of cellular ATP, thus reflecting cell viability after treatment.

### RNA extraction and quantitative RT-PCR

Total RNA was isolated from cells using Trizol (Invitrogen). 1μg of RNA were reversed transcribed with M-MuLV Reverse Transcriptase (New England Biolabs) and a mix of oligo dT and random hexamers for priming. Real time analysis was implemented using a PCR master mix (750mM Tris-HCL, 200mM (NH_4_)_2_SO_4_, 0,1% Tween20, 3mM MgCl_2_, SYBR Green 1:80.000, 0,2mM dNTPs, 20U/ml Taq Polymerase, 0,3M Trehalose, 0,3μM primer). Primers are listed in Supplemental Table 3. The RT-PCR was performed with a two-step protocol (2min at 95°C preheating, 40cycles at 95°C for 15s, followed by 60°C for 1min) monitoring SybrGreen fluorescence. Data were normalized to reference gene 36B4. Relative gene expression was calculated by using the ΔΔCt method.

### Immunohistochemistry

Clinical samples of invasive ductal carcinoma were classified by a board-certified pathologist. Samples of normal breast tissue removed for cosmetic reasons were used as references. Immunohistochemistry (IHC) was performed on a BenchMark XT autostainer (Ventana Medical Systems). Human tissue was first fixed in PBS containing 4% formaline and then embedded in paraffin. 1.5μm sections were cut and treated with boric-acid/EDTA buffer for antigen-retrieval. Primary antibodies, anti-Bcl-xL clone 54H6, 1:500, (rabbit monoclonal; Cell Signaling Technology) and anti-N-cadherin clone 6G11, 1:200, (mouse monoclonal; Dako) and OptiView DAB IHC detection kits (Ventana) were used. Bcl-xL specificity was verified using MSP RAS cells treated with siRNA against Bcl-xL [data not shown]. IHC stainings were scored by a pathologist. Digital quantification was performed by whole slide scanning (ScanScope XT) and ImageJ image analysis software (http://rsb.info.nih.gov/ij). 8bit intensity values were obtained using color deconvolution [38]. Deconvolution was used to demonstrate colocalisation in brightfield double-IHC. Intensity values are depicted as inverted and normalized greyscale. Statistical testing was performed using chi squared test on R version 2.15.2.

Xenograft tumors were analysed for autophagy and proliferation. Paraffin-embedded tumor tissue sections (5 μm) were deparaffinized and rehydrated. For antigen retrieval, sections were heated in 10 mM Citrat buffer pH 6.0 (Ki67) and 0.01M EDTA pH 7.5 (LC3A/B), respectively. After blocking with 3% hydrogen peroxide (only for Ki67) and 10% FCS/5% BSA in TBS, the following antibodies were applied: LC3A/B (Abcam) and Ki67 (BD Pharmingen) (all 1:50) overnight at 4°C. For immunohistochemical staining, sections were incubated with Envision-peroxidase (DAKO, Hamburg, Germany), and diaminobenzidine was used as chromogen. Finally, tissue sections were counterstained with hemalum. Sections were analysed with a BX60 microscope and the CVII camera using the Cell F software (Olympus). The percentage of Ki67 positive cells were determined by whole slide scanning (100x) with Image J image analysis software using color deconvolution, calculating the ratio between nuclear and Ki67 staining.

### Immunofluorescence

For immunofluorescence staining of xenograft tumors, sections were first exposed to primary antibody and then incubated with secondary anti-rabbit Cy3 antibody 1:1000 (Sigma) for 2h. DAPI/Vectashield was used as mounting medium. Images were obtained with a laser scanning microscope (Fluoview 1000, Olympus) and the FW 10-ASW software. For negative controls, blocking solution was used in place of the primary antibody. The percentage of LC3 positive cells was determined by whole slide scanning (100x) with AlphaView (ProteinSimple), calculating the ratio between Dapi and LC3 staining.

### Gene expression analysis

For comparison of mRNA expression levels of 24^+^ HMLE and MSP cells, a whole genome microarray was performed. For every cell line, three independent samples were prepared. Total RNA was isolated using Trizol and delivered to the transcriptome analysis laboratory (TAL) Göttingen. TAL controlled the RNA quality, measured the concentration and used 200ng of total RNA for reverse transcription into cDNA. cDNA was transcribed into antisense RNA with a master mix containing the sample cDNA, rNTPs, T7 RNA polymerase and Cy3-CTP. Antisense RNA was hybridized to the microarray slide. Array slides were read out by laser application, measuring the fluorescence intensity of the Cy3 dye. For analyses, data of each cell line were combined and the MSP data were analysed relatively to the 24^+^ HMLE data. The threshold of differentially regulated mRNA expression in MSP cells compared to 24^+^ HMLE was set to 2, and the induction was calculated as log2 values. Positive values indicate an up-regulation and negative ones a down-regulation of gene expression in MSP cells compared to 24^+^ HMLE.

### Statistical analysis

If not stated otherwise, data are shown as mean ± S.E.M. Unpaired Students T-test was used for the calculation of P values. n values in figure legends indicate the number of independent replicates.

## SUPPLEMENTARY FIGURES AND TABLES


