# Supplementary material for: Bcl-xL mediates therapeutic resistance of a mesenchymal breast cancer cell subpopulation
Source: Oncotarget. 2014 Nov 28;5(23):11778–91. doi: 10.18632/oncotarget.2634 (PMC4322974; doi:10.18632/oncotarget.2634)
Supplement: Supplementary file 1 [file oncotarget-05-11778-s001.pdf]

SUPPLEMENTARY FIGURES AND TABLES

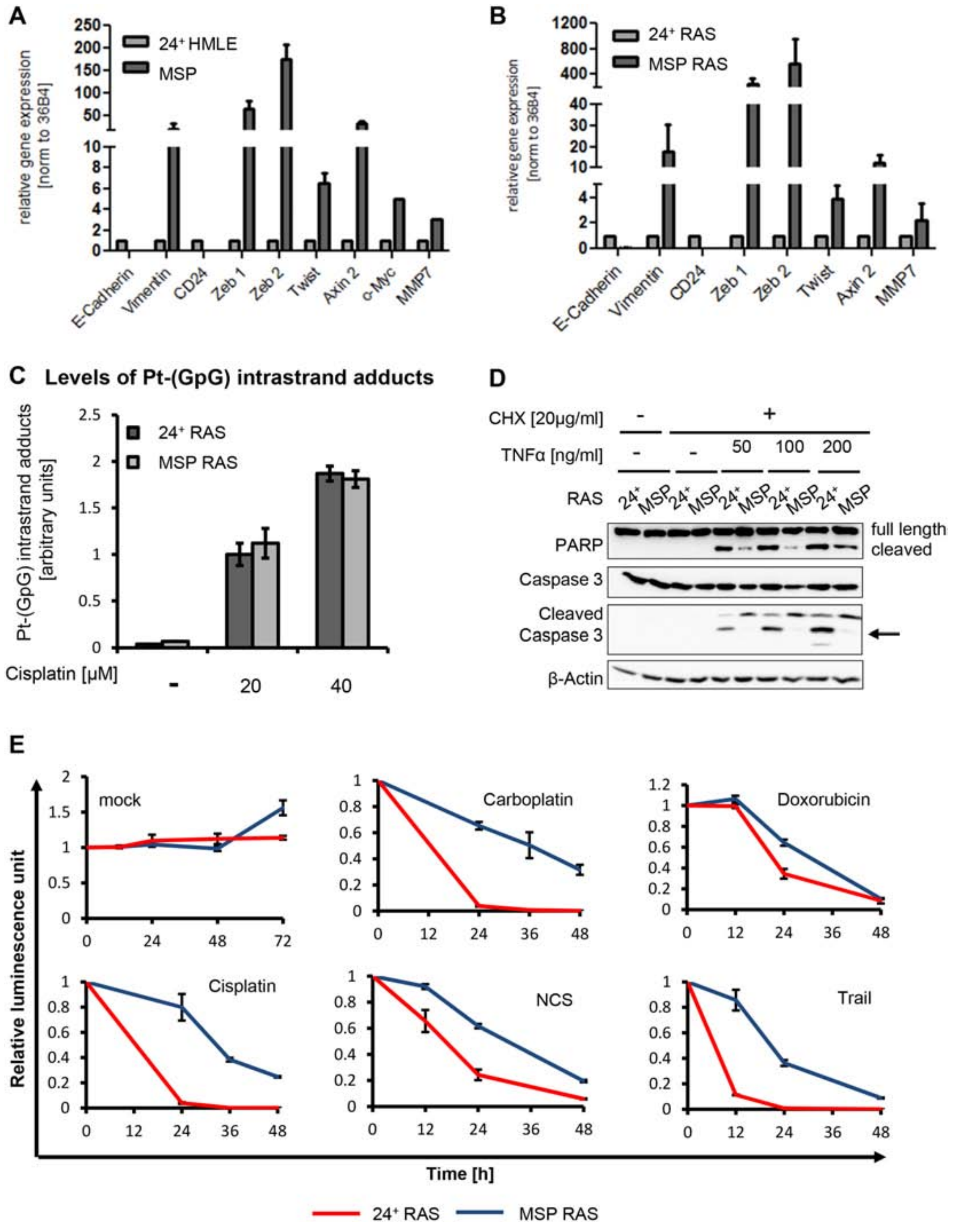

Supplementary Figure S1: Quantification of EMT marker gene expression in HMLE and HMLE RAS cells. (A, B) mRNA levels of the indicated genes were analysed in HMLE (A) and HMLE RAS cells (B) by qRT-PCR. mRNA levels were normalized to 36B4 mRNA. Columns and error bars represent the mean ± S.E.M. of $n = 3$. **(C)** 24^+ RAS and MSP RAS cells show a similar degree of DNA platination upon incubation with cisplatin. HMLE RAS cells were treated with 20µM or 40µM cisplatin for 16h or left untreated. DNA-platinum adducts were quantified by immuno-slot-blot assay. Columns represent the mean ± S.E.M. of $n = 3$. **(D)** HMLE RAS cells were treated with the indicated concentrations of TNFα in the presence of 20µg/ml cycloheximide for 6h. Cell lysates were analysed by immunoblotting. β-Actin was used as loading control. **(E)** HMLE RAS cells were treated with 1.5mM Carboplatin, 40µM Cisplatin, 500nM Doxorubicin, 500ng/ml Neocarzinostatin or 100ng/ml Trail in the presence of 20µg/ml CHX for 12h to 48h. DMSO treated cells were used as control. Cell viability upon treatment was determined by assessing the ATP concentration in cell lysates using a luciferase assay.

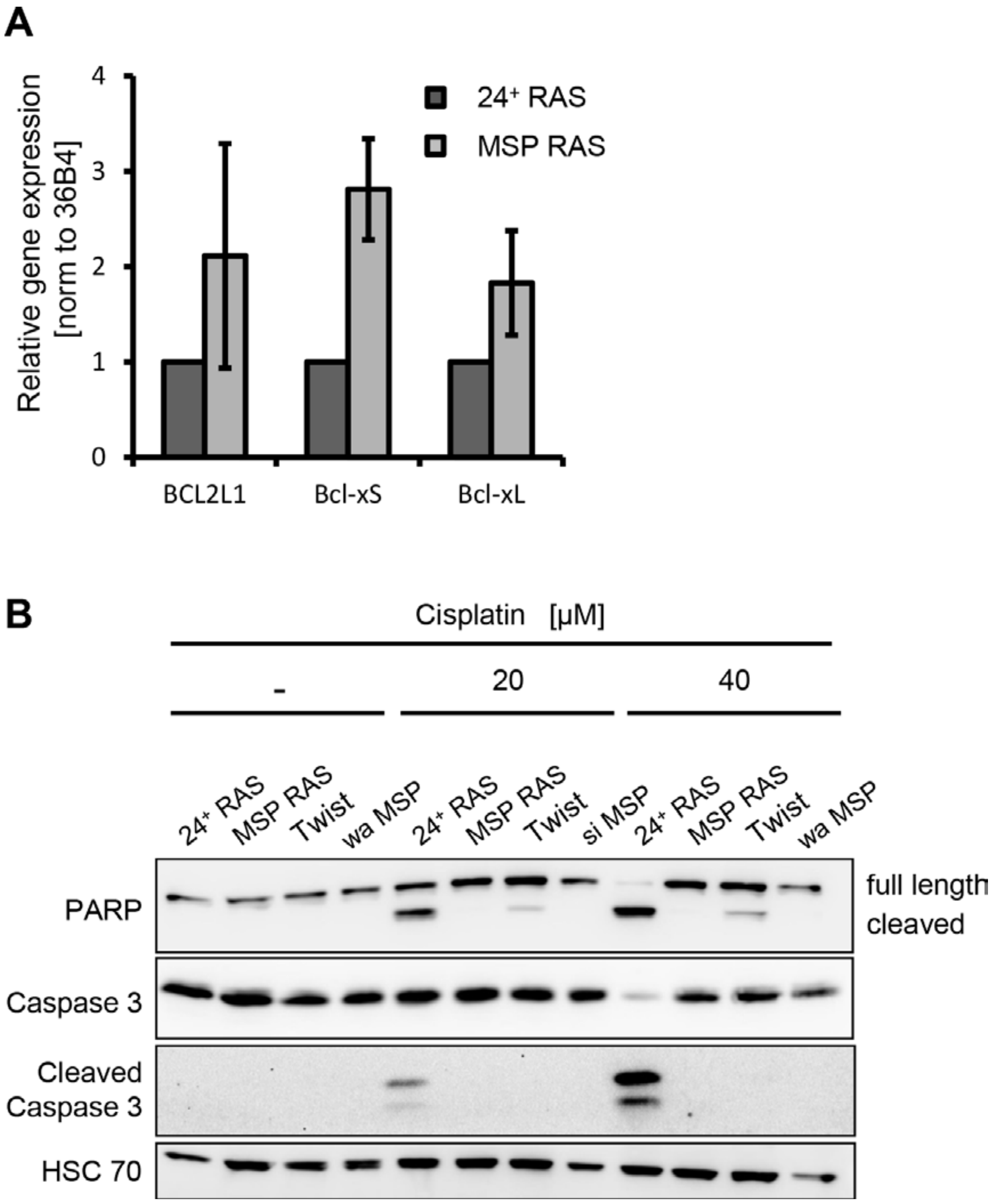

Supplementary Figure S2: EMT causes overexpression of the anti-apoptotic protein Bcl-xL and apoptosis-resistance. **(A)** mRNA of HMLE RAS cells was analysed for *BCL2L1* variants by qRT-PCR. mRNA levels were normalized to 36B4. Data show mean ± S.E.M. of $n = 5$. **(B)** HMLE RAS, HMLE Twist and wa MSP cells (cf. Fig. 2G) were treated with 20µM and 40µM Cisplatin for 16h. Cell lysates were analysed for PARP cleavage and cleaved Caspase 3 as indicators for apoptosis by immunoblot. HSC70 was used as loading control.

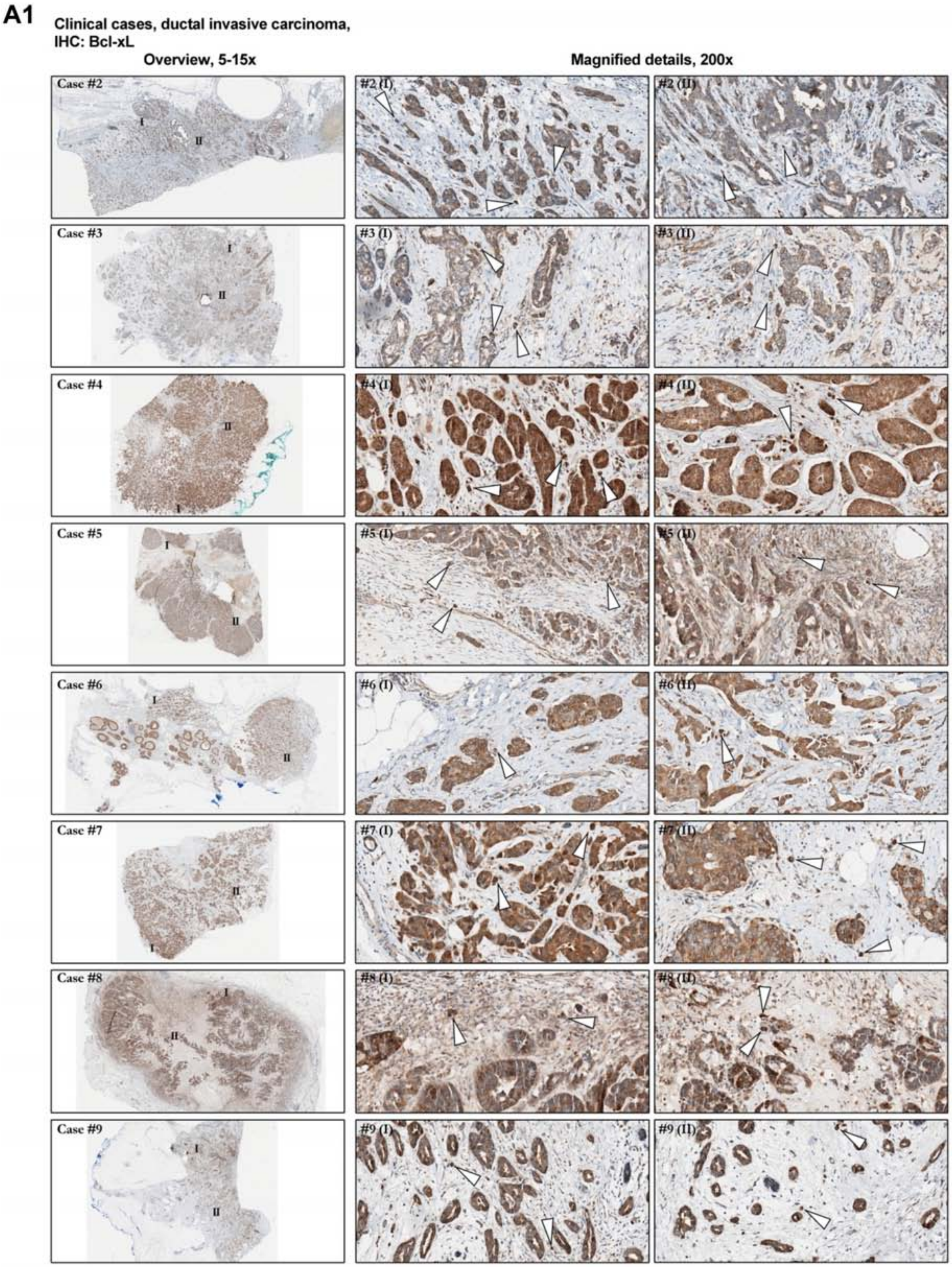

Supplementary Figure S3: Bcl-xL protein levels are increased in human breast cancer cells that are dispersed in the desmoplastic stroma. Human tissue, fixed in PBS-buffered formalin (4%), was embedded in paraffin. 1.5µm sections were treated with boric-acid/EDTA buffer for antigen-retrieval, followed by incubation with primary antibodies, secondary peroxidase-coupled antibodies, and diaminobenzidine (DAB).

(*Continued*)

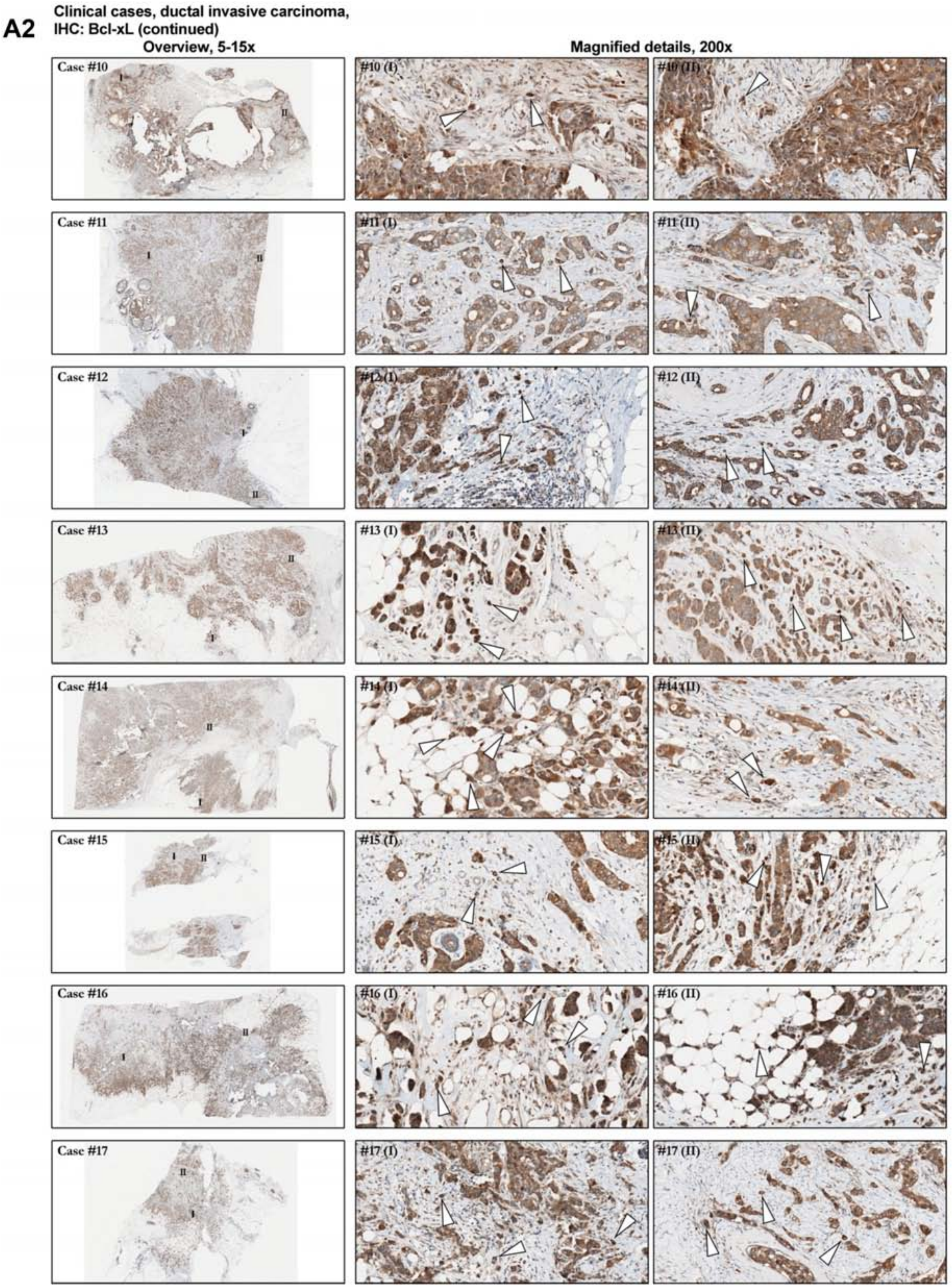

Supplementary Figure S3 (*Continued*): (A) Clinical cases, ductal invasive carcinoma, magnification 200x. Staining for Bcl-xL. Column 1: Overview, Columns 2-3: Magnifications of areas indicated in Column 1. Representative cells are highlighted (white arrows).

(*Continued*)

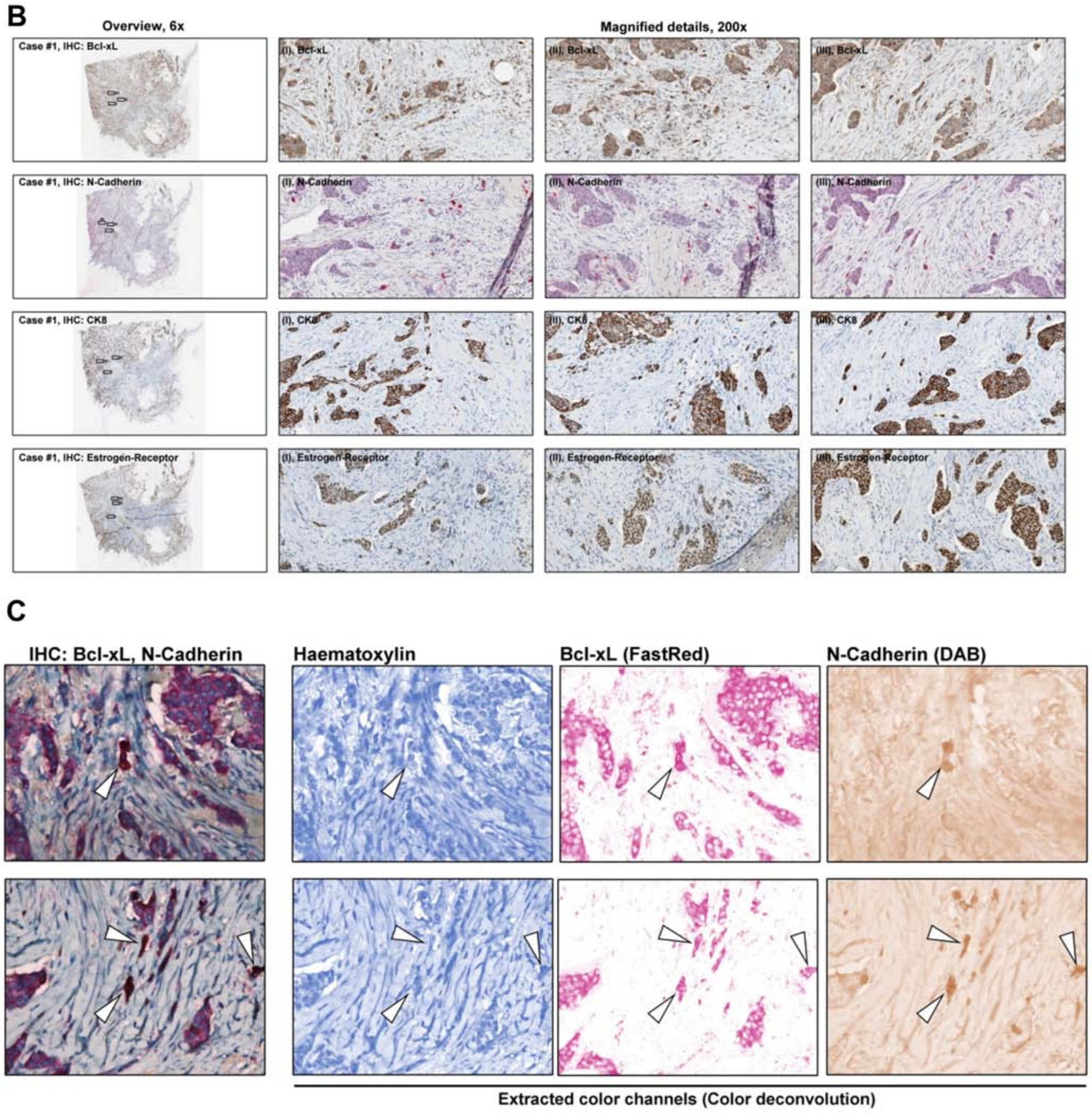

Supplementary Figure S3 (*Continued*): (B) Detailed staining for Bcl-xL, N-cadherin, CK8 and Estrogen Receptor in case #1. Column 1: Overview, Columns 2-4: Magnifications of areas indicated in Column 1. **(C)** Colocalisation of Bcl-xL and N-cadherin shown by color deconvolution in brightfield double-IHC.

(*Continued*)

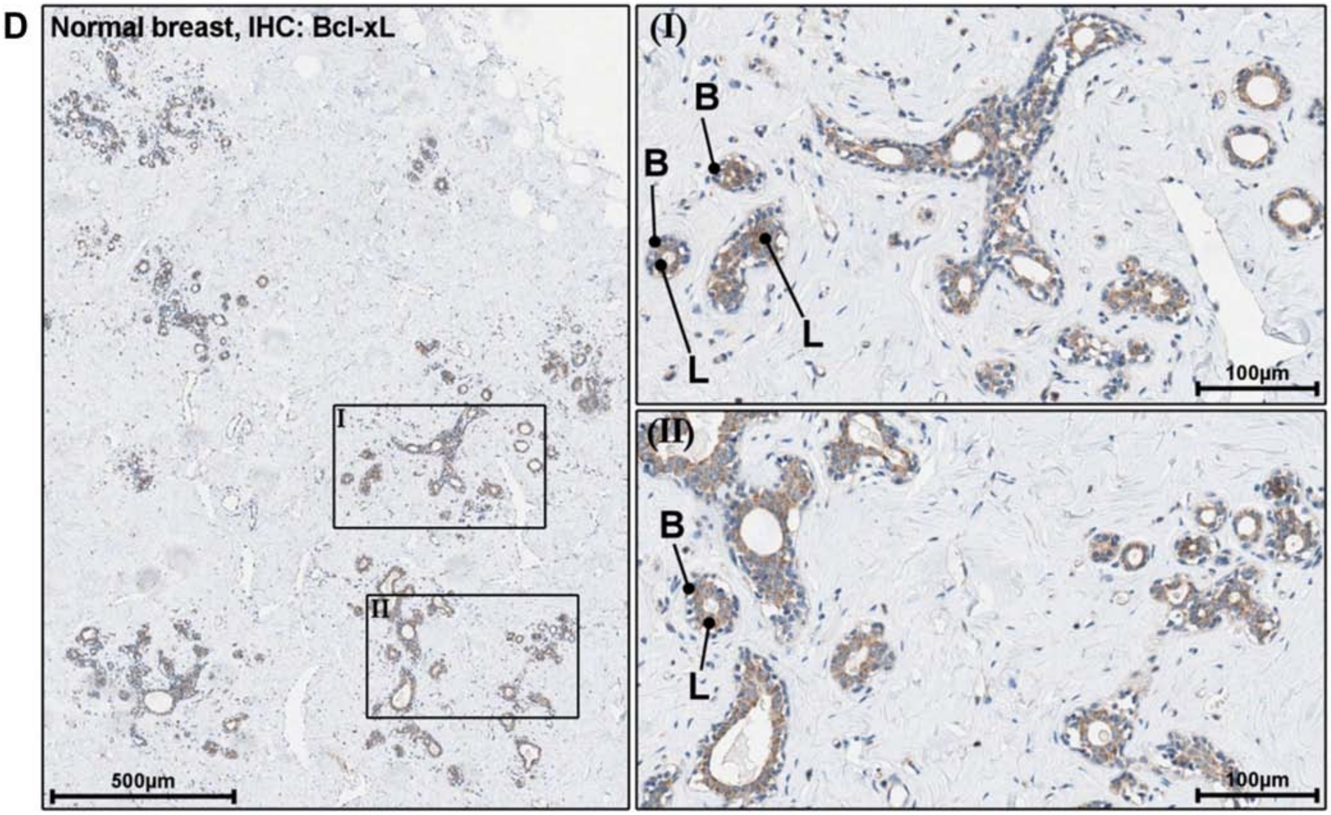

Supplementary Figure S3 (*Continued*): (D) Normal breast IHC for Bcl-xL. I and II are enlargements of the indicated areas. Bcl-xL is restricted to luminal cells (L) but not basal cells (B).

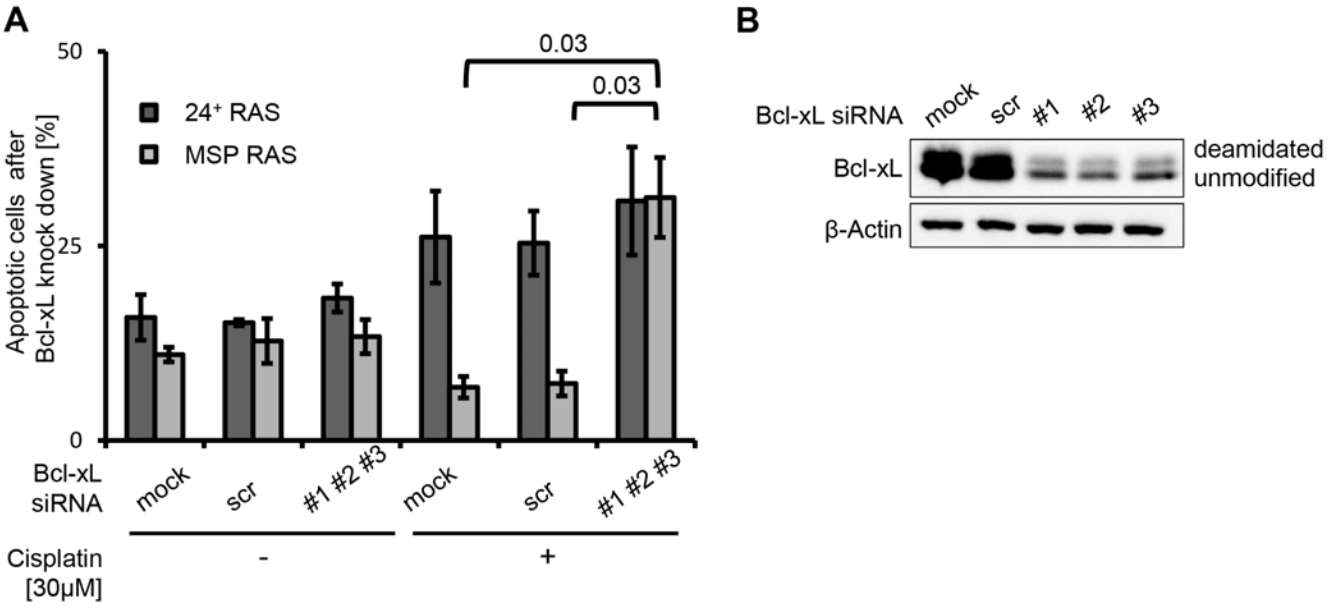

Supplementary Figure S4: Bcl-xL knock down sensitizes MSP RAS cells to cisplatin treatment. (A) HMLE RAS cells were depleted of Bcl-xL by siRNA and treated with 30µM cisplatin for 16h. Apoptotic cells were determined using flow cytometry, based on the labeling of activated caspases. Columns and error bars indicate the mean ± S.E.M. of *n* = 3. Student´s t-test was performed to indicate the P-value. **(B)** MSP RAS cells were depleted of Bcl-xL by siRNA. For determining the knock down efficiency cell lysates were analysed for Bcl-xL by immunoblotting. Note that the same blot was probed for Caspase 3, cleaved Caspase 3 and PARP in Fig. 4A. Therefore, β-Actin as loading control is used for Fig. 4A and Supplemental Fig.S4B.

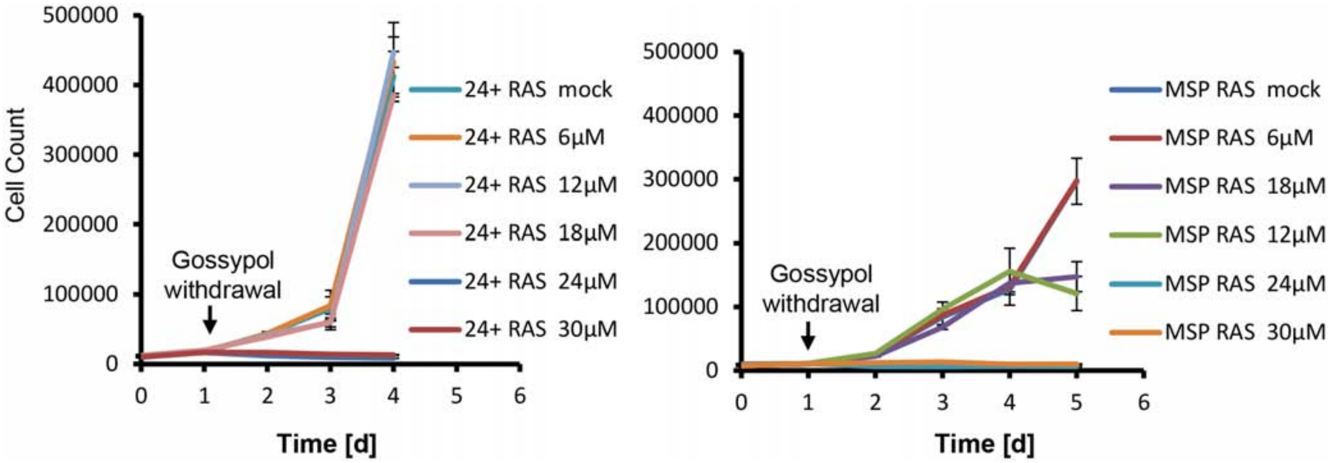

Supplementary Figure S5: Gossypol overcomes apoptosis resistance of MSP RAS cells. HMLE RAS cells were treated with gossypol, or with DMSO, for 24h as indicated, followed by the addition of fresh medium. Cell numbers were determined daily for 8d by microscopy and digital image analysis.

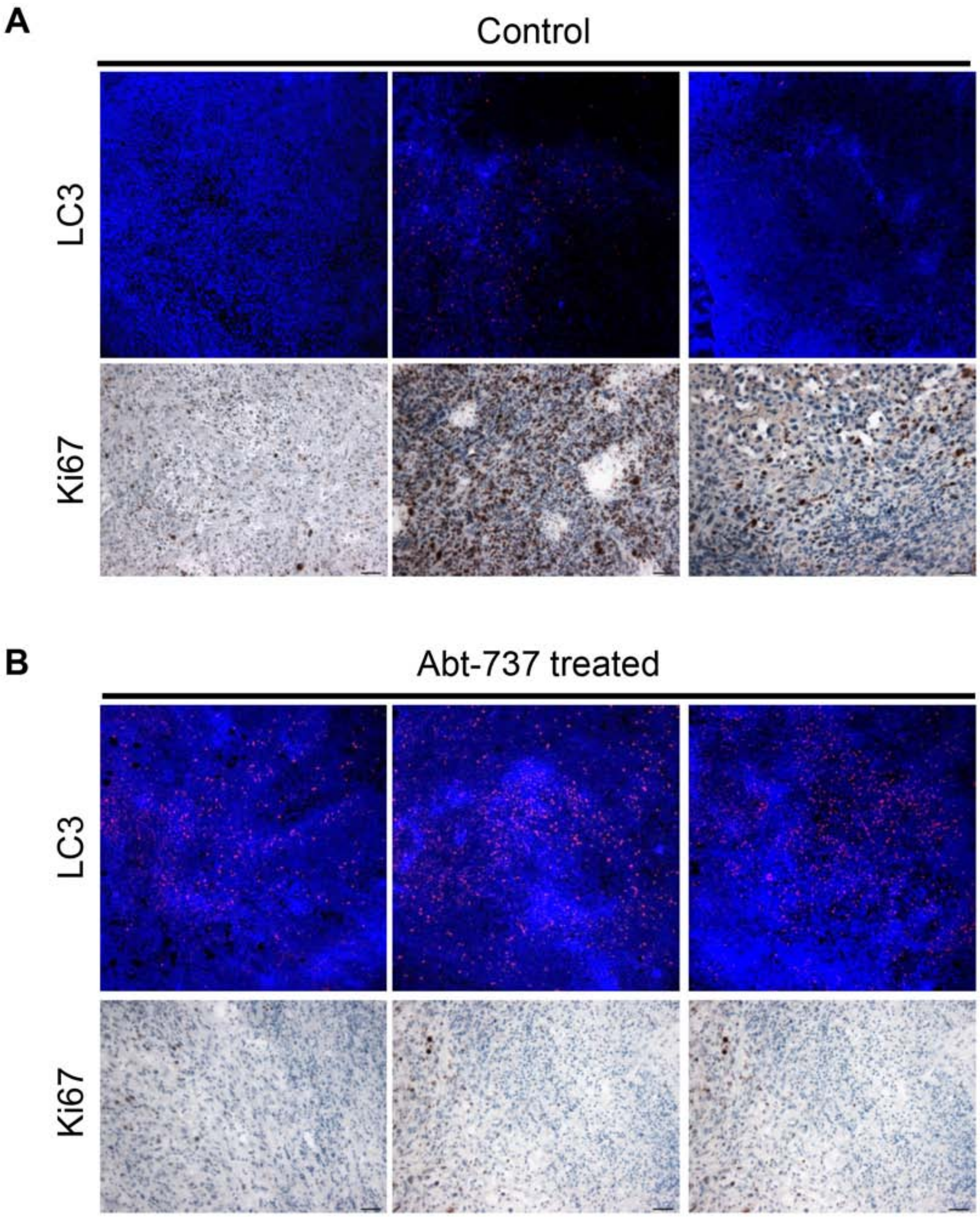

Supplementary Figure S6: BH3-mimetics prolong the survival of mice carrying MSP RAS xenografts. IHC and IF analysis of xenograft tumor tissue was performed for detection of Ki67 or LC3, respectively (cf. Fig. 6B). **(A)** Analysis of tumors from control mice. **(B)** Analysis for tumors from mice treated with Abt-737. 100x magnification.

Supplementary Table S2.1. Clinical cases of mammary ductal invasive carcinoma and presence of dispersed Bcl-xL positive cells (Summary)

		total (n)	DIC, no DBCs	DIC, with DBCs	% DBCs
	# Cases	56	39	17	30
	Age		67.5y (SD±13.4)	64y (SD±14)	
	Female		38	17	
Receptor status (IHC)	HR positive	25	12	13	52
	HR and Her2 positive	3	2	1	33
	Her2 positive and HR negative	12	10	2	17
	Triple negative	16	15	1	6
	presence of DCIS	26	14	12	46
	absence of DCIS	30	25	5	17

DIC= Ductal invasive carcinoma
DCIS= Ductal carcinoma in situ
DBCs= Dispersed Bcl-xL positive cells
HR= Hormone receptors, i.e. Proteinexpression of Estrogenrecetor, Progesteronreceptor or both
Her2= Proteinexpression of Her2/Neu receptor

Chi-squared test; DCIS and DBCs

	DBCs		
	absent	present	Pearson's Chi-squared test
DIC, DCIS absent	25	5	X-squared = 4.4186
DIC, DCIS present	14	12	p-value = 0.03555

Chi-squared test; Proteinexpression of Hormone receptors and DBCs

	DBCs		
	absent	present	Pearson's Chi-squared test
DIC, HR positive	14	14	X-squared = 8.4465
DIC, HR negative	25	3	p-value = 0.003658

Supplementary Table S3. Primers for quantitative real-time PCR

Target	fwd	rev
Axin 2	5′-TCCCCACCTTGAATGAAGAA-3′	5′-TGGTGGCTGGTGCAAAGA-3′
BCL2L1	5′-CAAGCGCTGAGGGAGGCAGG-3′	5′-GCCCTTTCGGCTCTCGGCTG-3′
Bcl-xL	5′-CCTTTTTCTCCTTCGGCGGGGC-3′	5′-GCCCTTTCGGCTCTCGGCTG-3′
Bcl-p1A	5′-CCTCTCCCGACCTGTGATA-3′	5′-AAAGTCAACCACCAGCTCCC-3′
Bcl-p1B	5′- ATGAAGGGGGATGTGGCC-3′	5′-AAAGTCAACCACCAGCTCCC-3′
Bcl-p2	5′-GTGAAGTATCTTGGAACCTAGACCCA-3′	5′- ATAGGGATGGGCTCAACCAGTCC-3′
Bcl-xS	5′-AGAGCTTTGAACAGGATACTTTTGTGGA-3′	5′-TGAGCCCAGCAGAACCACGC-3′
CD24	5′-ACCCAGCATCCTGCTAGACGCG-3′	5′-AGTTGGATTTGGGGCCAACCCAGA-3′
E-Cadherin	5′-CCTGGACGCTCGGCCTGAAG-3′	5′-ATAAGGCGGGGCTGTGGGGT-3′
MMP7	5′-GAGGAGCTCATGGGGACTC-3′	5′-CCATAGGTTGGATACATCACTGC-3′
Twist	5′-GGAGTCCGCAGTCTTACG-3′	5′-TCTGGAGGACCTGGTAGAGG-3′
Vimentin	5′-CGTGTATGCCACGCGCTCCT-3′	5′-TCGAGCTCGGCCAGCAGGAT-3′
Zeb1	5′-GCGCAGAAAGCAGGCGAACCC-3′	5′-CCCTTCCTTTCCTGTGTCATCCTCC-3′
Zeb2	5′-AACGGTCCTGCCTCCCGACA-3′	5′-AGTTCGCATGGACTCGGCGC-3′
36B4	5′-GATTGGCTACCCAACTGTTG-3′	5′-CAGGGGCAGCAGCCACAAA-3′
